# Health promotion interventions for community-dwelling older people with mild or pre-frailty: a systematic review and meta-analysis

**DOI:** 10.1186/s12877-017-0547-8

**Published:** 2017-07-20

**Authors:** Rachael Frost, Celia Belk, Ana Jovicic, Federico Ricciardi, Kalpa Kharicha, Benjamin Gardner, Steve Iliffe, Claire Goodman, Jill Manthorpe, Vari M Drennan, Kate Walters

**Affiliations:** 1 Department of Primary Care and Population Health, University College London, London, UK; 2 Department of Statistical Science, University College London, London, UK; 3 Department of Psychology, King’s College London, London, UK; 4 Centre for Research in Primary and Community Care, University of Hertfordshire, Hertfordshire, UK; 5 Social Care Workforce Research Unit, King’s College London, London, UK; 6 Centre for Health and Social Care Research, Kingston University & St George’s, University of London, London, UK

**Keywords:** Frailty, Pre-frailty, Systematic review, Health promotion

## Abstract

**Background:**

Mild or pre-frailty is common and associated with increased risks of hospitalisation, functional decline, moves to long-term care, and death. Little is known about the effectiveness of health promotion in reducing these risks. This systematic review aimed to synthesise randomised controlled trials (RCTs) evaluating home and community-based health promotion interventions for older people with mild/pre-frailty.

**Methods:**

We searched 20 bibliographic databases and 3 trials registers (January 1990 – May 2016) using mild/pre-frailty and associated terms. We included randomised controlled and crossover trials of health promotion interventions for community-dwelling older people (65+ years) with mild/pre-frailty and excluded studies focussing on populations in hospital, long term care facilities or with a specific condition. Risk of bias was assessed by two reviewers using the Cochrane Risk of Bias tool. We pooled study results using standardised mean differences (SMD) where possible and used narrative synthesis where insufficient outcome data were available.

**Results:**

We included 10 articles reporting on seven trials (total *n* = 506 participants) and included five trials in a meta-analysis. Studies were predominantly small, of limited quality and six studies tested group exercise alone. One study additionally investigated a nutrition and exercise intervention and one evaluated telemonitoring. Interventions of exercise in groups showed mixed effects on functioning (no effects on self-reported functioning SMD 0.19 (95% CI -0.57 to 0.95) *n* = 3 studies; positive effects on performance-based functioning SMD 0.37 (95% CI 0.07 to 0.68) *n* = 3 studies). No studies assessed moves to long-term care or hospitalisations.

**Conclusions:**

Currently the evidence base is of insufficient size, quality and breadth to recommend specific health promotion interventions for older people with mild or pre- frailty. High quality studies of rigorously developed interventions are needed.

**PROSPERO registration:**

CRD42014010370 (Review 2).

**Electronic supplementary material:**

The online version of this article (doi:10.1186/s12877-017-0547-8) contains supplementary material, which is available to authorized users.

## Background

Frailty is a transitional process of increasing vulnerability to adverse health outcomes and reduced functional reserves, in which persons have an increasing risk of functional decline, disability, falls, hospitalisation and death [1–3]. Estimates suggest that 10.7% of community-dwelling older adults in developed countries are frail [4] and 42% have the transitional state of pre-frailty, where an person has some frailty characteristics but is able to respond to injury, disease or stress with a chance of complete recovery [1].

Pre-frailty is commonly assessed using the Fried phenotype (presence of 1–2 of the following: slow gait speed, weakness, low energy, unintentional weight loss and low physical activity) [2]. The Fried phenotype is recommended for research as it provides more consistent prevalence estimates, though focusses solely on physical domains [4]. Other definitions include the Rockwood Clinical Frailty Scale (persons are classified through clinical judgement of disability level into eight categories, from very fit to very severely frail) [3], the cumulative deficits model (a continuous score of frailty according to the accumulation of symptoms, diseases, disabilities and laboratory findings) [5] and less frequently used performance-based or self-reported assessments [6].

Though less vulnerable than frail older adults, pre-frail people are at higher risk than robust adults of greater frailty, hospitalisation, falls, worsening disability and mortality [2]. As the number and proportion of people aged 60 and over are increasing substantially in populations worldwide [7], this is triggering concerns about increased health and social care costs. Older people with mild or pre-frailty are more likely to transition back to a robust state than those who are frail [8], and so health promotion represents an important opportunity to prevent decline and dependence, and to potentially make gains in health and reductions in disability and need for care.

However, we lack evidence as to which health promotion interventions may be clinically and cost-effective in reducing adverse outcomes in older people with mild or pre-frailty, previous research having largely focussed on the most frail older people with the highest level of disability or illness [9, 10]. A recent scoping review of frailty prevention interventions by Puts et al. found some positive effects for a range of interventions (mostly exercise-, nutrition- or comprehensive geriatric assessment-based) on reducing frailty prevalence or the number of frailty markers [11]. However, this review included frailty status only as an outcome, for which there are some concerns regarding a lack of evidence of measurement properties apart from construct validity and the level of change which may be considered clinically meaningful [12]. Guidance on frailty trials has suggested that functioning may be a more relevant outcome to study in frailer populations [13].

Puts et al.’s review [11] and another review protocol by Apostolo et al. [14] also combine frail and pre-frail populations. Whilst this provides evidence of which interventions could have an impact upon a change in frailty state, interventions such as preventative home visits appear to be more effective in younger populations and those at lower risk of mortality [9], and there is an increased likelihood of reversing pre-frailty as opposed to frailty [8]. A review of interventions targeted specifically at pre-frail populations and focussing on physical functioning and a wider range of outcomes therefore contributes valuable evidence as to what works specifically within pre-frail populations. Within this present review we aimed to synthesise evidence from randomised controlled trials (RCTs) to evaluate the effectiveness of home- and community-based health promotion interventions on functioning and frailty in older people with mild or pre-frailty.

## Methods

We used systematic review methods as outlined by Higgins et al. [15]. This review is registered in PROSPERO (CRD42014010370, Review 2) and is reported according to PRISMA guidelines [16].

### Search strategy and selection criteria

We included English language studies with (1) community-dwelling older people (mean age > =65), with mild frailty identified through a validated frailty scale which contained an intermediate classification between frail and robust (e.g. pre-frailty on the Fried phenotype [2], mild frailty on the Rockwood Clinical Frailty Scale [3], the modified physical performance test [17], the Electronic Frailty Index [18]), including studies reporting subgroup analyses for a mildly frail subsample, (2) home- or community-based health promotion interventions (i.e. interventions that enable people to improve or increase control over their health [19]), (3) any comparison group, (4) outcome of self-reported or observed physical functioning, as the recommended indicator of quality of life and health service utilisation in frail populations [13], frailty or associated components (e.g. gait speed, muscle strength, physical activity), quality of life (considered a valuable secondary outcome in frailty trials [13]), hospital admissions, moves to long term care, (5) randomised controlled parallel-group or crossover trials.

We excluded studies (1) in populations without a clear or validated definition of pre-frailty (e.g. ‘functionally limited’), pre-frail populations within a restricted range of health conditions and hospital or long-term care home populations to increase generalizability to the wider population, (2) inpatient interventions, (3) studies using biological markers only as outcomes (e.g. markers of inflammation, muscle composition) as single biomarkers are considered inadequate predictors of frailty [20], (4) other study designs (e.g. cohort, qualitative, non-randomised trials). We excluded studies combining an early frailty population with a frail or robust sample for analysis. We contacted all corresponding authors of papers that reported the presence of a pre-frail group but did not report outcomes for this group in their paper and were not also excluded on other grounds (*n* = 10/14), but did not receive a response.

We searched the following databases (Jan 1990-May 2016): MEDLINE, MEDLINE in Process & Other Non-Indexed Citations, EMBASE, Scopus, Social Science Citation Index, Science Citation Index Expanded, Cochrane Database of Systematic Reviews, Cochrane Central Register of Controlled Trials, Cochrane Effective Practice and Organisation of Care Group, NHS Health Economic Evaluation Database, Cochrane Methodology Register, Cochrane Groups, Database of Abstracts of Reviews of Effects, PsycINFO; Cumulative Index to Nursing and Allied Health Literature, Evidence for Policy and Practice Information Centre Register of Health Promotion and Public Health Research (Bibliomap), Sociological Abstracts, Social Care Online, and Applied Social Sciences Index and Abstracts.

Databases were searched in all fields using “mild frail*”, “early frail*”, “transitioning to frail*”, “transitionally frail*”, “pre frail*”, prefrail* and pre-frail*, combined with the Boolean operator “OR”. Title, abstract and keyword limiters were used in Web of Science and Scopus. Medical subject heading terms were not used as there are currently no specific pre-frailty terms. We searched the NIHR Health Technology Assessment database, the UK Clinical Research Network Portfolio Database and ClinicalTrials.gov and located full texts where possible if the trial details matched our inclusion criteria. We reviewed the reference lists of relevant systematic reviews and emailed authors of relevant conference abstracts and protocols to obtain study outcome reports where available. We performed forward citation tracking and reference list screening for all included studies.

Titles and abstracts of articles were screened by one researcher (CB, AJ or RF) against the agreed inclusion and exclusion criteria. Full text copies were screened by two reviewers (AJ and CB or RF). Disagreements were resolved through discussion between reviewers and with a third reviewer (KW) where necessary.

### Data extraction and quality assessment

Data were extracted by RF on study design, sample size, inclusion and exclusion criteria, frailty definition, intervention and control details, outcomes assessed and results. Two independent reviewers (RF and CB or AJ) assessed risk of bias using the Cochrane Risk of Bias tool [21], with disagreements resolved by discussion among reviewers. The Cochrane Risk of Bias tool assesses six domains of bias that may affect the internal validity of a study as high (likely to have an effect on the results or conclusions), unclear (insufficient data to make a judgement) or low. The domains include selection bias (how the randomisation sequence was generated and concealed), performance bias (how participants were blinded to allocation), detection bias (how outcome assessors were blinded to allocation), attrition bias (whether any outcome data were incomplete, excluded from the analysis or related to allocation), reporting bias (whether some outcomes reported in the protocol were not reported in the paper) and any other reason a study may be at risk of bias [22]. Studies were rated as low risk of bias in participant blinding if they used an active control but this was not considered a key domain for the overall risk of bias in the trial, as a usual care control is often appropriate within exercise trials. Risk of bias was undertaken for descriptive purposes, not as an inclusion or weighting criterion for meta-analysis.

### Synthesis

We undertook meta-analysis for outcomes in which more than one study assessed the same outcome for a similar intervention, and narratively synthesised other outcomes and studies. When a study used more than one assessment of the same construct (e.g. two balance measures), we selected the most comprehensive, valid and reliable measure for an older population through reviewing the associated literature for each measure. As studies used differing lengths of intervention and follow up periods, we synthesised outcomes collected at the post-intervention endpoint. There were too few studies to examine long-term follow up, though data from these were narratively summarised. Where more than one report was published, the report with the most complete data was used as the primary report and data extracted from this. We contacted the authors where possible when further unpublished data (e.g. standard deviations) were needed for inclusion in meta-analysis.

We calculated standardised mean differences (SMD) for post-intervention endpoint scores for studies using different measures of the same construct. Where only change scores were available, we approached authors or reported these scores narratively as change scores cannot be synthesised meaningfully with SMD endpoint scores [15]. As studies contained some differences in exercise dose and populations, we combined studies using random effects as we expected meta-analysis to produce a mean effect across studies [23]. We used the I^2^ statistic to assess heterogeneity for each outcome [15], although study numbers were insufficient to explore potential sources of heterogeneity.

Where studies compared more than one exercise intervention to a control (e.g. power training, strength training and control), we statistically combined the mean and SD from both groups as advocated by Higgins et al. [15]. Where crossover designs were used, we included data from only the first period of the trial (obtained from the authors), as exercise interventions are likely to have lasting effects and no washout period was used in the included trial [24]. We did not assess publication bias as the number of studies was too small to meaningfully determine if funnel plots were asymmetrical [25].

## Results

**Fig. 1:**
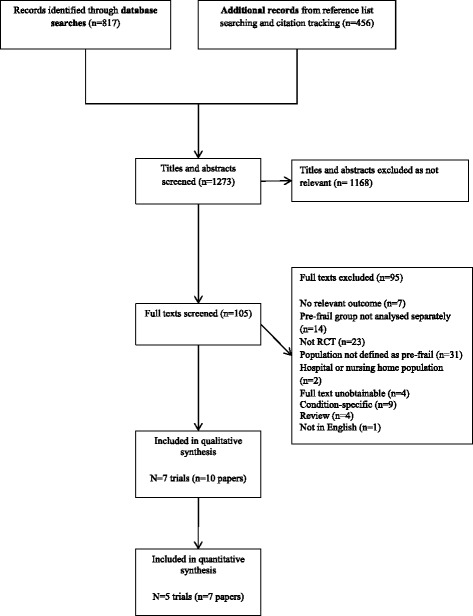
PRISMA flow diagram of studies included in the review

Of 1273 unique references identified through searches 10 papers (7 RCTs reporting on 10 interventions) were eligible for inclusion in this review (Fig. 1, Table 1 and Additional file 1).

**Table 1:** Description of included studies

Study ID country	N randomised	Intervention	Control	Intervention period Follow up period	Outcomes assessed	Main findings
Binder 2002 [26, 33] USA	119	Balance and strength exercises	Flexibility home programme	9 months 3, 6 and 9 months	Performance-based physical functioning Self-reported functioning Balance Muscle strength Quality of life	Significant improvements in exercise group vs control in observed and self-reported functioning. Some differences in balance, muscle strength and quality of life subscales.
Brown 2000 [27] USA	87	Balance and strength exercises	Home range of motion exercises	3 months 3 months	Performance-based physical functioning Muscle strength Balance Gait speed	Significant improvements in observed functioning and balance in exercise group and mixed improvements in muscle strength across different muscle groups. No differences in gait speed.
Daniel 2012 [30] USA	23	1. Wiifit exercise 2. Seated exercise	Usual activity	15 weeks 15 weeks	Self-reported functioning Physical activity Timed up and go	No statistical comparison between groups; within-group improvements in some aspects of the Senior Fitness test for both exercise groups (between group changes not assessed). Wii group increased physical activity.
Drey 2012 [28, 34] Germany	69	1. Power training 2. Strength training	Usual activity	12 weeks 12, 24 and 36 weeks	Performance-based physical functioning Self-reported functioning Muscle strength	Significant differences in SPPB score changes at 12 weeks between each exercise intervention and control, but effects not maintained at 24 or 36 weeks. No differences in muscle strength or self-reported functioning at 12, 24 or 36 weeks.
Kwon 2015 [29] Japan	89	1. Strength and balance training + nutrition 2. Strength and balance training alone	General health education sessions	12 weeks 3 and 9 months	Gait speed Balance Muscle strength	No significant differences between any groups in the majority of observed functioning and quality of life domains.
Lustosa 2011 [31, 35] Brazil	32	Resistance exercise	Usual activity	10 weeks 10 and 20 weeks	Gait speed Timed up and go Muscle strength	Significant improvements in observed function and muscle power in exercise group when both exercise phases (*n* = 32) compared to first control phase (*n* = 16), no differences between groups at the end of the first period.
Upatising 2013 [32] USA	87	Telemonitoring	Usual care	12 months 6 and 12 months	Frailty state	No statistical comparison for pre-frail group; slightly higher transitions to non-frail and frail in usual care.

### Description of included studies

We reviewed four parallel group RCTs [26–29], one pilot RCT [30], one randomised crossover trial [31] and one secondary analysis of a RCT [32]. Sample sizes ranged from 23 to 194 (total *N* = 506 participants randomised and *N* = 485 contributed data to the synthesis) and all contained either two (*n* = 5) or three (*n* = 2) trial arms. For three studies, additional publications were used as secondary sources of data [33–35].

Age inclusion criteria ranged from 60+ to 78+ (mean age range: 72–83 years). Four studies recruited pre-frail older adults or women [29–31, 34] defined according to the Fried phenotype [2] (with or without modifications) and two recruited mildly frail populations according to the modified physical performance test [26, 27]. One study divided participants into frailty level according to the Fried phenotype and data were extracted for the pre-frail subsample [32].

Four RCTs evaluated single interventions – three of group exercise [26, 27, 31] and one of telemonitoring [32]. Three RCTs evaluated two interventions, either two exercise interventions [30, 34] or exercise and nutrition vs exercise [29]. Further details of each intervention are provided in Table 2. All exercise interventions were supervised by trained instructors, exercise physiologists or physiotherapists and conducted in a clinical, laboratory or community setting. Sessions ranged from 45 –60 min one to three times a week, over a 12–36 week period. Comparators included usual activity [30, 31], usual activity plus an invitation to two physical activity and nutrition lectures [34], monthly general health education sessions [29] and a low intensity flexibility home exercise programme [26, 27]. In one study all participants received vitamin D for an 8 week run-in phase prior to randomisation [34].

**Table 2:** Description of interventions included in the review

Study reference	Duration	N (post-int)	Frequency and duration of sessions	Intervention content	Professional and setting	Adherence
Drey 2012 [28, 34]	12 weeks. All received 8 weeks Vitamin D supplementation prior to randomisation, stratified by baseline level.	18	2 × 60 minute sessions per week	*Power training (PT):* Walking, balance exercises and upper and lower body progressive explosive resistance training using a “Bodyspider” machine.	Trained instructors in an exercise room in a clinical setting.	Attendance: mean 68%, median (range) 88% (25–96)
		20	2 × 60 minute sessions per week	*Strength training (ST):*Walking, balance exercises and upper and lower progressive resistance training using a “Bodyspider” machine.	Trained instructors in an exercise room in a clinical setting.	Attendance: mean 80%, median (range) 92% (83–96)
		22	n/a	*Control:* maintain current activity and invited to 2 physical activity and nutrition lectures.	n/a	n/a
Daniel 2012 [30]	15 weeks	7	3 × 45 minute exercise sessions per week	*Wii-fit:* Nintendo Wii basic games (e.g. bowling, tennis and boxing) plus a weight vest carried out in small groups.	Study staff, location not reported.	86% attendance
		7	3 × 45 minute exercise sessions per week	*Seated exercise:* Progressive exercises to increase lower and upper body strength and flexibility, seated or using chairs for support, plus walking and stretching.	group led by certified fitness instructor at study site	86% attendance
		5	n/a	*Control:* usual physical activity and exercise.	n/a	n/a
Binder 2002 [26, 33]	9 months	66	3 sessions per week (duration not reported)	*Exercise:* 3 progressive 3mo phases of balance, flexibility, coordination, reaction speed and strength exercises.	Group exercise sessions supervised by 3 exercise physiology technicians at university indoor exercise facility.	100% - Participants were required to undertake all sessions before progressing to the next stage. Intervention completed in 422 ± 85 days.
		49	2–3 times per week, plus monthly exercise class to enhance adherence.	*Control:* low-intensity flexibility exercise programme.	Unsupervised home programme	Home participants completed the programme in 250 ± 65 days. Compliance recorded on a calendar but not rigorously monitored.
Kwon 2015 [29]	12 weeks	26	1 × 1 hour exercise plus 1 × 2–3 h cooking class per week	*Exercise training and nutrition:* Exercise: group strength and balance training Nutrition: cooking class including food preparation, nutrition guidance, cooking instructions, cooking practice, eating together and tidying up, focussing on protein- and vitamin D-rich foods.	Exercise supervised by a health fitness trainer (+1 physician and 2 assistants) at research centre, with materials for home practice. Cooking class run by 4 dieticians.	Not reported
		25	1 × 1 hour per week	*Exercise training:* group strength and balance training	Supervised by a health fitness trainer (+1 physician and 2 assistants) at research centre, with materials for home practice.	Not reported
		28	Monthly	*Control:* group general health education sessions (physical training for falls prevention and urinary incontinence, dietary guidance for healthy ageing)	Research centre, provided by health fitness trainer, physician and dietician.	n/a
Lustosa 2011 [31, 35]	10 weeks per group	16^a^	3 × 1 hour per week	*Exercise*: Small group lower limb resistance exercises.	Supervised by a physiotherapist (setting not reported)	Not reported
		16	*n/a*	Control: continue normal activities of daily life without training	*n/a*	n/a
Brown 2000 [27]	3 months	48	3 exercise sessions per week	*Exercise:* 22 progressive flexibility, balance, body handling skills, speed of reaction, coordination and strength group exercises.	Outpatient rehabilitation fitness centre (professional not reported).	100% - Participants required to complete all sessions prior to outcome assessment.
		39	Home frequency not reported, monthly supervised session	*Control:* home range of motion exercises, plus on-site exercise once a month.	Home (unsupervised). Supervising professional not reported.	“Self report by the participants and the significant improvements in range-of-motion values indicate that home exercises were done by subjects” (p.964)
Upatising 2013 [32]	12 months	102	n/a	*Telemonitoring:* Equipment installed in participant’s home and blood pressure, pulse, oxygen saturation, blood glucose and weight measured as per an individualised protocol.	Data reviewed by healthcare team, with person or physician contact as needed	Not reported
		103	n/a	*Usual care:* face-to-face visits, phone services and home health care as needed.	Usual services	n/a

^a^ *n* = 32 in original paper, *n* = 16 in meta-analysis

Three studies evaluated self-reported functioning as a secondary outcome, using the Functional Status Questionnaire (34 items assessing activities of daily living (ADL), instrumental ADL, psychological, social, work, social interaction domains) and the Older American Resources and Services instrument (social and economic resources, mental and physical health, ADLs) [26], the Later Life Function and Disability Index (LLFDI) Function subscale (assesses difficulty with 32 functional tasks) [30] and the Short Form LLFDI (a 15-task LLFDI subscale) [34].

Three studies assessed performance-based physical functioning across multiple domains as their primary outcome. These used either the Short Physical Performance Battery (SPPB) [34], a composite measure of lower extremity functioning involving an assessment of balance, gait speed and chair stands [36] or the modified Physical Performance Test [26, 27], a test of upper and lower extremity function consisting of nine tasks (e.g. lifting a book, putting on and removing a coat) [17]. Daniel 2012 and Kwon 2015 evaluated physical performance using three or more domains reported separately (Senior Fitness test and handgrip strength, gait speed and balance) [29, 30]. Kwon 2015 reported change scores only for all outcomes and so could not be included in the meta-analysis [15]. We emailed the authors for further data but received no response.

Four studies assessed gait speed [27, 29, 31, 34] (three with sufficient endpoint data for meta-analysis), three balance [26, 27, 34] and two mobility (timed up and go) [30, 31]. Muscle strength, evaluated using a variety of methods, was assessed in five studies [26, 27, 29, 31, 34] and four contained sufficient data on knee extensor strength for meta-analysis. Of outcomes that could not be meta-analysed, two studies assessed quality of life as a secondary outcome using the Short Form-36 [26, 29], one study assessed physical activity using the Community Healthy Activities Model Program for Seniors questionnaire [30] and one assessed frailty state transitions only [32]. No studies evaluated hospital admissions or care needs as an outcome.

### Risk of bias

**Fig. 2:**
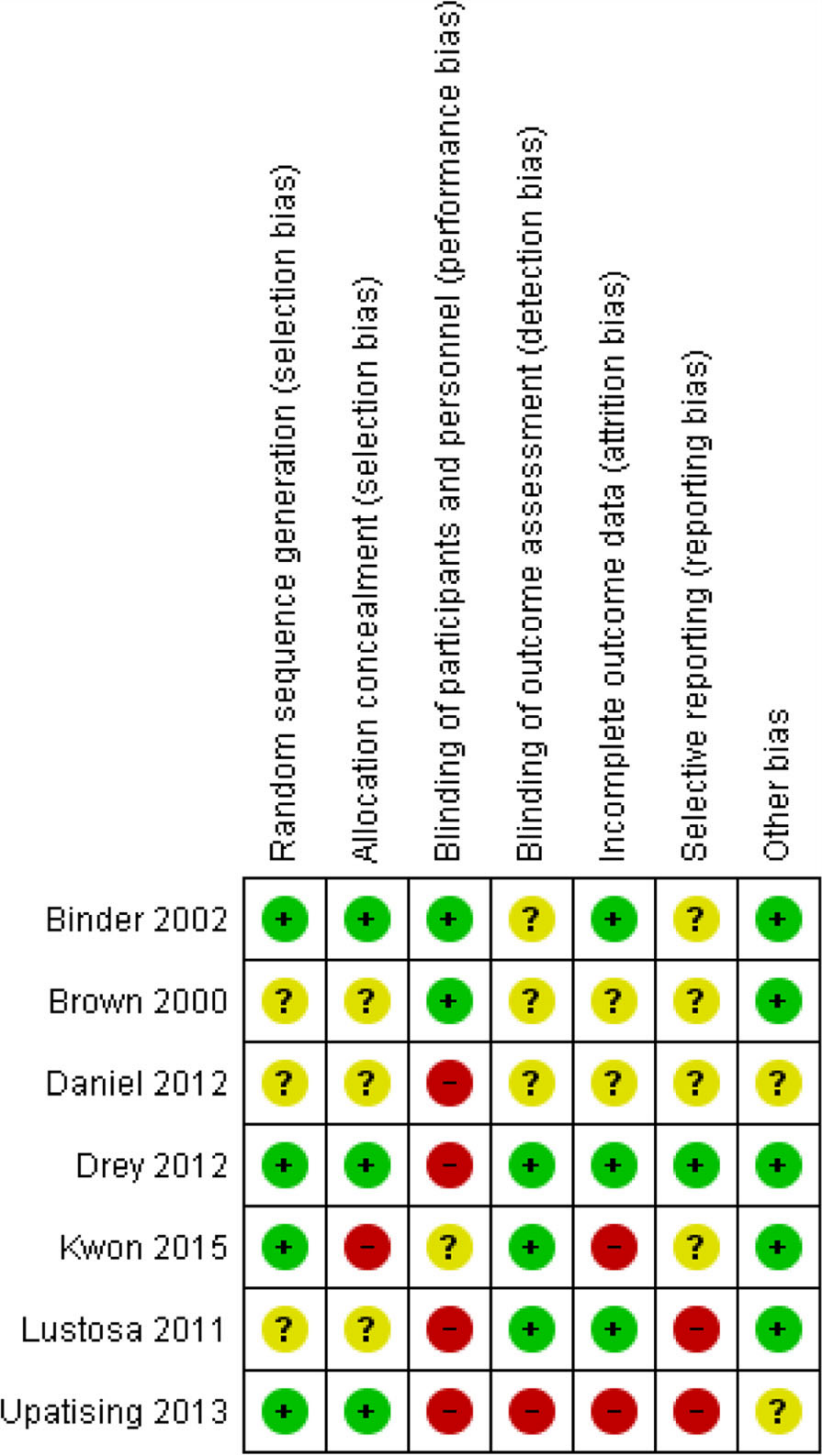
Risk of bias graph for included studies

Across each risk of bias domain, bias risk was generally low or unclear (see Fig. 2). Participant blinding had a high risk of bias in four studies [30–32, 34], but as stated above usual care is often considered an appropriate control for health promotion interventions and so this was not considered a key domain. Two studies were at high risk of bias from attrition [29, 32] and selective reporting [31, 32], whilst one study had inadequate allocation concealment [29]. Regarding individual trials, one study was at low risk of bias across all key domains [34] (see Fig. 2). For two studies, the overall risk of bias was unclear, with some domains rated as low risk [26, 27]. In one study all key domains were of unclear bias risk due to poor reporting [30], whilst three studies were at overall high risk due to high risk of bias in one [31] or two or more [29, 32] key domains.

### Evidence synthesis

**Fig. 3:**
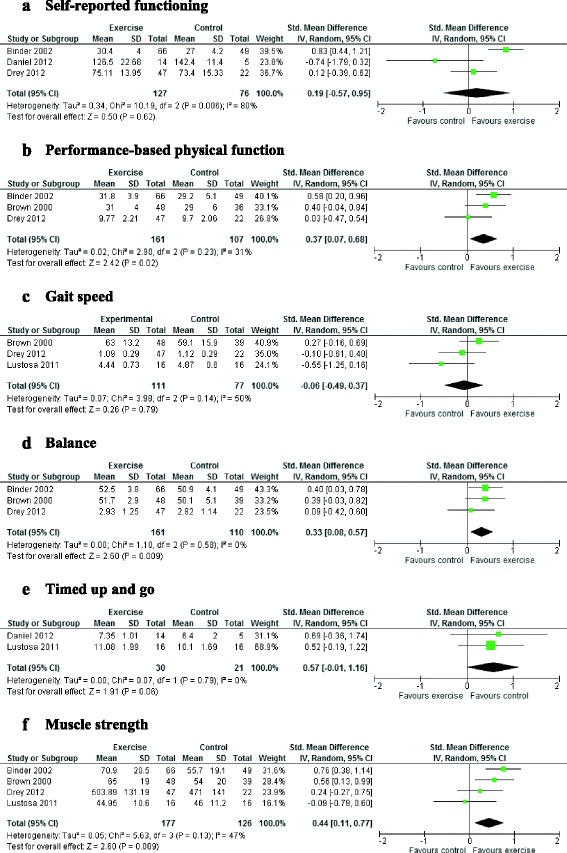
Forest plots of the effects of health promotion interventions in mild or pre-frailty

Six studies evaluated community-based group exercise interventions in older adults with pre- or mild frailty compared to a usual activity or a control home flexibility programme and reported post-intervention endpoint data. Of these six, five contained sufficient intervention endpoint data for meta-analysis (one included change scores only) and so these were considered suitable for meta-analysis where more than one study assessed the same outcome type. Fig. 3a-f summarise the forest plots for each outcome.

#### Self-reported functioning

We found no evidence for group exercise on self-reported functioning (Fig. 3a, n=3, SMD 0.19 (95% CI -0.57 to 0.95)). However, most included trials were fairly small, with substantial heterogeneity in outcomes. The only study to show evidence of effect was the largest, with the longest intervention period (9 months), but this also found no difference between groups in use of human or technological assistance in ADLs using the Older American Resources and Services instrument [26].

#### Multidomain performance-based physical functioning

Overall, group exercise interventions had a significant and beneficial effect on physical functioning immediately post-intervention compared to usual care or a flexibility home control exercise programme (Fig. 3b, n=3, SMD 0.37 (95% CI 0.07 to 0.68)). Strongest effects were found in Binder 2002, in the longest exercise programme (9 months) [26]. Drey 2012 found that exercise effects were not maintained 12 or 24 weeks after the intervention ended [34].

#### Objective single domain measures

Results from single domain functioning measures were mixed (see Figs. 3c-f). We found no effects upon gait speed (*n* = 3 studies, SMD -0.06 (CI -0.49 to 0.37), moderate heterogeneity) or timed up and go speed (*n* = 2 studies, SMD 0.57 (CI -0.01 to 1.16), low heterogeneity), but significant differences in muscle strength (*n* = 4, SMD 0.44 (CI 0.11 to 0.77)), moderate heterogeneity) and balance (*n* = 3 studies, SMD 0.33 (CI 0.08 to 0.57), low heterogeneity)). Within one study that could not be included in the meta-analysis, no significant between group differences were reported between the exercise and nutrition, exercise only or control groups in handgrip strength, balance or gait speed at 3 months (post-intervention) or 9 months, apart from an increase in grip strength in the exercise group at 3 months [29].

#### Other outcomes

Comparing telemonitoring to usual care in a secondary analysis, slightly fewer people transitioned from pre-frail to non-frail (9/35 (26%) vs 12/38 (32%)) or to frail (3/35 (9%) vs 6/38 (16%)) over 6 months [32]. Binder 2002 and Kwon 2015 found no significant differences in overall quality of life in exercise and exercise and nutrition interventions [26, 29], whilst in Daniel 2012 WiiFit exercise caused an increase in self-reported physical activity [30].

## Discussion

We identified 7 RCTs evaluating interventions designed and tested specifically for older people with mild or pre-frailty in community settings. On the basis of the available evidence, no specific interventions can currently be recommended for promoting health in mild or pre-frailty. Most studies focussed on a single domain (exercise/muscle strength). Group exercise interventions had some positive effects on functioning, but these were mixed and based on small, low quality studies.

Similarly mixed effects upon physical performance and wide variation in intervention content and delivery have also been found in a meta-analysis of exercise interventions for frail older adults [10], suggesting that exercise alone may be insufficient for improving functioning. Apart from one study incorporating a cooking class as a nutritional intervention component and one telemonitoring study, neither of which produced significant effects, no studies were found of other interventions targeted at pre-frail populations. However, cohort and cross-sectional studies suggest that mood, cognitive state and loneliness can be important determinants of frailty and functional decline [37–40] and stakeholders have argued for broader interventions addressing a wider range of frailty components such as these [11]. Combined interventions have shown additive effects in some RCTs in combined pre-frail and frail populations [41–43], though others have found mixed effects for multidimensional interventions such as comprehensive geriatric assessment) [11, 44–47]. This suggests further investigation of effective combinations of components is needed.

Though some behaviour change techniques have been associated with improved physical functioning in a review of frailty health promotion interventions, interventions in this paper were largely poorly reported and lacked a theoretical basis for behaviour change [48]. A theoretical basis can identify the mechanisms by which behaviours are changed, and provide key information as to what to include, modify or combine within future interventions [49]. This suggests that comprehensive developmental work is needed prior to evaluating interventions to identify the most effective components for future services to address a broader range of dimensions, such as psychological, cognitive, social and socioeconomic effects [1]. A multitude of potentially effective interventions may therefore remain untested among this population.

### Strengths and limitations of included studies

Included studies were generally small and low quality, with short follow-ups and focussed on observed physical functioning outcomes. Studies at higher risk of bias were typically small and favoured the control arm or null effects, though studies at lower risk of bias still showed mixed effects upon the outcomes included in this review. Though physical functioning is a good frailty indicator [13], hospitalisation, long-term care moves, mortality rates and intervention cost-effectiveness were not assessed in any study, despite being key targets and considerations in health promotion interventions. Activities of daily living support needs were assessed in one study but data were not reported in the paper [26]. A few studies were excluded as they did not clearly report frailty status for pre-frail and frail older people separately.

Mild and pre- frailty definitions were inconsistent across trials. Whilst we only included studies using a validated method of identifying mild or pre-frailty and five trials used the Fried phenotype (recommended for research for the more consistent estimates of pre-frailty [4]), three made minor to major modifications of these criteria. We included studies which suggested participants were ‘mild to moderately frail’ as the included participants’ mean scores indicated they fell within a mildly frail range on the modified physical performance test. These two studies tended to show stronger effects upon outcomes. Inconsistent definition of frailty and pre-frailty is a widely recognised issue within frailty research [6, 50]. When further research is available, stratifying meta-analyses by pre-frailty definition would be helpful to identify the best way of targeting the pre-frail population most likely to benefit from interventions.

Some trials had limited generalisability as they excluded people with a history of orthopaedic surgery or fracture, mild cognitive impairment, chronic obstructive pulmonary disease or conditions where vigorous exercise was contraindicated; many of which are common in early frailty. A small number appeared to recruit fairly healthy older people (e.g. Kwon et al. defined a “slow gait speed” as <1.52 m/s), limiting the generalisability of these results to populations who are frailer, e.g. those defined as “mildly frail” on the Rockwood scale typically require help with instrumental activities of daily living [3] suggesting a much higher dependency level. Different age groups (e.g. 60+, 78+) were also targeted, which may add greater variability in results as older age groups have a greater likelihood of frailty. However, within this review there were too few trials to determine whether or how these factors may have had an impact upon outcomes.

### Strengths and limitations of the review

To our knowledge, this review is the first to focus solely on interventions developed for and tested among older populations with mild or pre- frailty. Mild and pre-frailty are not always defined or labelled well within databases, particularly prior to the development of the Fried phenotype, which may mean that some relevant studies were not included. However, we performed thorough citation tracking, reference list screening and searched ongoing databases to identify further relevant studies – for example Brown 2000 [27] used mild-moderate frailty criteria but described their participants as frail rather than pre-frail. We traced protocols located in database searches and on trials registries to identify the full text or contacted the authors where possible for unpublished data.

There were few available studies for meta-analysis and most outcome estimates had moderate heterogeneity, which could arise from exercise content or the outcome measures used, limiting the conclusions that could be drawn. Poor reporting hindered risk of bias assessment and inclusion in meta-analysis for some studies. Though we only included studies published in English, which may have limited the literature, we reviewed studies carried out in a range of countries.

Pre-frailty and frailty prevention is a rapidly evolving field. Our searches of ongoing trials revealed a number of potentially relevant ongoing studies, tabulated in Additional file 2. Since the conclusion of this review, two potentially relevant RCTs have been published. One found that 10 weeks of L-carnitine supplementation in pre-frail Malaysian older adults had some effects on Frailty Index score and handgrip strength compared to placebo, but not on other physical performance measures [51]. One combined nutritional screening and exercise intervention reduced the odds of Spanish pre-frail older adults transitioning to frailty, but had no other effects on physical performance outcomes [43]. Both therefore support the mixed findings within this review, but do not affect the review conclusions as neither would be eligible for inclusion in the meta-analysis. However, this suggests that nutritional interventions may be a promising component to investigate in future pre-frailty health promotion intervention evaluations.

### Implications for practice

At present, there is insufficient evidence to recommend any specific interventions or intervention components for promoting health or preventing worsening frailty state, hospitalisation or moves to long-term care in mild or pre-frailty. Interventions targeted at and developed with this population are currently sparse. Group exercise may produce some benefits to physical functioning, but findings for similar outcomes (e.g. self-reported and observed functioning) were contradictory, suggesting that conclusions may be open to change in the future. Other interventions (e.g. nutrition, mood, telemonitoring) also lack sufficient evidence at present.

### Implications for research

Despite the large population of pre-frail older adults and the high potential for promoting health and preventing decline in this population, interventions and evaluations targeted at this population are currently lacking. Ongoing studies (see Additional file 2) are still largely based on physical activity interventions, sometimes with a nutritional component. Included papers typically did not report consulting pre-frail older people regarding intervention design and provided little detail about the intervention development and rationale. Stakeholder consultations suggest that older people and professionals favour a multidimensional approach to health promotion in pre-frailty, including psychosocial dimensions [11, 52]. Involving pre-frail older people in intervention design is recommended in health promotion interventions [53], has previously led to significant changes to interventions to maximise their acceptability and relevance [54] and has been reported to be valuable in developing interventions targeted at frailty prevention [55]. Future involvement could be achieved through use of co-design processes to develop or refine intervention content and delivery or through consulting older adults as to the relevant outcomes for a service.

Future RCTs need to be high quality, adequately powered and use validated and consistent definitions of pre-frailty. The long-term effects of interventions need to be assessed and, where possible, other clinically relevant outcomes such as hospitalisation rates, social care needs and cost-effectiveness should also be included.

## Conclusion

Currently, no specific interventions can be unreservedly recommended for health promotion in older adults with mild or pre-frailty as the evidence base is small and of limited quality. Though group exercise may produce limited effects on physical functioning, evidence from broader, well-developed interventions addressing a wider range of frailty components and clinically relevant outcomes (e.g. hospitalisation) is needed.

## Additional files

Additional file 1: Table of detailed study characteristics. (DOCX 29 kb)

Additional file 2: Table of ongoing studies identified in this review. (DOCX 19 kb)

## Abbreviations

ADL: Activities of daily living
CI: Confidence interval
LLFDI: Late-life function and disability index
PRISMA: Preferred reporting items for systematic review and meta-analysis
RCT: Randomised Controlled Trial
SMD: Standardised mean difference
SPPB: Short physical performance battery
