# Supplementary material for: Health promotion interventions for community-dwelling older people with mild or pre-frailty: a systematic review and meta-analysis
Source: BMC Geriatr. 2017 Jul 20;17:157. doi: 10.1186/s12877-017-0547-8 (PMC5520298; doi:10.1186/s12877-017-0547-8)
Supplement: Supplementary file 1 — Table of detailed study characteristics. (DOCX 29 kb) [file 12877_2017_547_MOESM1_ESM.docx]

| **Study reference** | **Design, country** | **Participants** | **Outcomes analysed** | **Summary of findings** |
| --- | --- | --- | --- | --- |
| Binder 2002 [26, 33] | 2 group RCT (3:2 allocation)  USA | n=115 provided post-intervention data (119 randomised)  *Inclusion:* 78+yrs, mild-moderate frailty *Exclusion:* medical condition contraindicating vigorous exercise, neuromuscular disorders unlikely to improve with exercise, chronic use of corticosteroids, immunosuppressants, androgen-, oestrogen- or progestin-containing compounds, cigarette use within previous year, cancer diagnosis within previous year, sensory impairments interfering with following test instructions, significant cognitive impairment  83yrs (4), 60F/55M  Frailty definition: mPPT score 18-32 plus either difficulty with >=2 IADLs/1 ADL or VO_2peak_ 10-18ml/kg/min)  Baseline frailty: average mPPT score 28.4(4.7) (exercise) and 28.3(5.9) (control) | 3, 6 and 9 months.  1. Modified physical performance test (mPPT) (Primary outcome) 2. Activities of daily living (ADL) questionnaires (Primary outcome) a) Older American Resources and Services instrument b) Physical function subscale of Functional Status Questionnaire (FSQ) 3. Peak oxygen uptake (VO_2peak_, graded treadmill walking) (primary outcome)  4. Range of motion of hip, ankle, knee and shoulder (goniometry) 5. Balance a) Functional reach test b) Balance beam c) Single limb stance time d) Berg balance instrument 5. Short Form-36 6. Geriatric depression scale 7. Maximal voluntary muscle strength (Cybex isokinetic dynamometry) | Significant improvements in exercise group vs control in modified PPT score (29.2 vs 31.8 at test 4 groupxtest p=0.02), VO_2peak_ (15.2 vs 17.4 at test 4, groupxtest p=0.0001) and FSQ score (27.0 vs 30.4 at test 4, groupxtest p=0.01). No significant changes on Older American Resources and Services instrument ADL scale (data not reported in paper), but some changes in balance, muscle strength and health Short Form-36 subscale. No significant differences between groups for other outcomes. |
| Brown 2000 [17] | 2 group RCT  USA | n=84 provided post-intervention data (n=87 randomised) *Inclusion:* sedentary, >78yrs, living independently but with difficulty, PPT score 18-32.  Exclusion: PPT score >32 or =<17 83yrs (4), 57%F/43%M  Frailty definition: mPPT score 18-32  Baseline frailty: mPPT scores Exercise 29(4), Control 29(6) | 3 months. Primary outcome not specified 1. Physical Performance Test 2. Strength (lower: Cybex isokinetic dynamometer, upper: Micro-Fet dynamometer) a. Knee extensors and flexors b. ankle plantar and dorsiflexors c. shoulder flexion and abduction d. elbow flexion e. grip strength f. hip extension and abduction 3. Range of motion (goniometric measures) a. Passive shoulder flexion b. shoulder external rotation c. hip flexion with the knee extended  d. hip internal rotation e. knee flexion f. ankle dorsiflexion g. trunk rotation h. distance from fingertips to floor while bending i. hip flexor tightness 4. Balance  a. Static - Romberg test b. Dynamic - balance beam, obstacle course, fast gait speed c. Berg balance test  5. Gait analysis (pressure-sensitive foot switches to collect gait velocity, stride length, cadence, swing, stance time, double support time and percentage of gait cycle spent in each phase) 6. Coordination and response speed  a. Purdue peg board  b. Response time  7. Sensation a. Light touch and pressure (monofilaments pressed against plantar surface of toes and heel) b. Proprioception (tuning fork placed on foot. Scored if felt for 5seconds or more) | Significant improvements in mPPT from 29(4) to 31 (4) in exercise compared to home group (29(6) to 29(6), 2x2 ANOVA, p<0.05).Some significant changes in knee extensors and flexors and shoulder abductors but no other changes. Flexibility increased in both groups (no significant differences). Significant improvements in balance in the exercise group, but no significant changes in gait apart from preferred walking cadence. No changes in coordination or response time and no differences in sensation (both groups showed mild sensory loss). |
| Daniel 2012 [30] | 3 group pilot RCT  USA | N=19 provided post-intervention data (n=23 randomised)  *Inclusion*: 65+yrs, pre-frail (Fried criteria) *Exclusion:* not reported 77yrs (5.3), 14F/9M  Frailty definition: Fried phenotype (no further details)  Baseline frailty: 100% pre-frail | 15 weeks. Primary outcome not specified.  1. Senior Fitness Test  a) chair stands b) timed up and go c) arm curls d) sit and reach e) step 2 f) back scratch g) 6 minute walk 2. Community Healthy Activities Model Programme for Seniors  3. Activities-specific confidence scale 4. Late life function and disability index – function total, disability frequency, disability limitations | Within-group improvements in some aspects of the Senior Fitness test for seated exercise and Wii groups. Between group changes not assessed. Increase in energy expenditure for Wii group (Community Healthy Activities Model Programme for Seniors) and reduction in disability frequency (Late life function and disability index) across all groups. |
| Drey 2012 [28, 34] | 3 group RCT  Germany | n=69* provided 3mo data (n=69 randomised)  *Inclusion:* independent community-dwelling older adults aged 65-94, pre-frail (Fried criteria) *Exclusion:* depression (Geriatric Depression Scale>5), dementia (Mini-mental state examination<25), Body mass index >35, taking immunosuppressants, history of kidney stones, sarcoidosis, plasmacytoma, chronic obstructive pulmonary disease, inflammatory bowel disease, angina pectoris, history of cancer, current attendance at muscle training Control 76yrs, 73%F/27%M  Power training 78yrs, 67%F/33%M Strength training 77yrs, 70%F/30%M  Frailty definition: Fried phenotype, with unintentional weight loss self-reported rather than directly measured  Baseline frailty: 100% pre-frail | 12, 24, 36 weeks  1. Primary outcome: Short Physical Performance Battery (SPPB) 2. Sit-to-stand transfer power 3. Short Form Late Life Function and Disability Instrument 4. Appendicular lean mass | Significant differences in SPPB score changes at 12 weeks between each power training and control (+0.9points (CI 0.48-2.73), p=0.004) and strength training and control (+1.0points (CI 0.44-2.58), p=0.005), but not power vs strength training (Kruskal Wallis p=0.301). Effects were not maintained at 24 or 36 weeks. No differences in sit-to-stand power or Short Form Late Life Function and Disability Instrument between groups at 12, 24 or 36 weeks. |
| Kwon 2015 [29] | 3 group RCT  Japan | N=79 provided 3 month data (n=89 randomised) *Inclusion:* community-dwelling women aged 70+, pre-frail (modified Fried criteria)  *Exclusion*: serum albumin >=4.5mg/dL, serious musculoskeletal conditions, taking vitamin D or calcium supplements.  76.8yrs (range 70-84), 100%F  Frailty definition: 2 Fried criteria: muscle weakness (handgrip strength in lowest quartile at baseline (=<23kg)) and slow gait speed (lowest quartile at baseline =<1.52m/s).  Baseline frailty: 100% pre-frail | 3, 9 months 1) Physical performance a) Muscle strength (handgrip strength, Smedley’s Hand Dynanometer) b) Balance (stork stand time with eyes open) c) Walking (usual walking speed over 5m) 2) Health-related quality of life (8 domains of Short Form-36) | No significant differences between groups in physical performance apart from improved handgrip strength in exercise group at 3 month (not maintained at 6month). No significant differences between groups in quality of life apart from role emotional score at 3 months (not maintained at 6 month) in exercise+nutrition group. |
| Lustosa 2011 [31,34] | 2 group randomised crossover trial  Brazil | n=32 provided post-intervention data** (n=32 randomised) *Inclusion*: community-dwelling women aged 65+, pre-frail (Fried criteria) *Exclusion*: men, orthopaedic surgery or history of lower limb fracture, unable to walk without an aid, neurological conditions, acute musculoskeletal inflammatory conditions, already performing physical activity >=2x weekly, active neoplasia in the last 5 yrs, using drugs with a broad immune system action, cognitive alterations (based on Mini-mental state examination).  Exercise 72yrs (4), 100%F  Control 72yrs (3.5), 100%F  Frailty definition: Fried phenotype (no further details)  Baseline frailty: 100% pre-frail | 10, 20 weeks 1. Functional performance (primary outcome***)  a) Timed up and go  b) 10 metre walk test (6m are timed) 2. Muscle strength of knee extensors (isokinetic dynamometer Byodex System). | Both exercise phases (n=32) compared to first control phase (n=16) in paper. Significant improvements in functional performance (Timed up and go F=9.54, p=0.01, 10 metre walk test F=3.80 p=0.01) in exercise training group and muscle power at 180 degrees/s (tending toward significant at 60 degrees/s). |
| Upatising 2013 [32] | Secondary analysis of Tele-ERA RCT  USA | n=87 out of 194 with complete frailty data used in per-protocol analysis (n=205 randomised).  *Inclusion*: 60+, score of 16+ on elder risk assessment  *Exclusion:* living in a nursing home; dementia; score of <=29 on the Kokmen short mental status test, unable to give informed consent; inability to use telemonitoring equipment. 80.4yrs (8.3), 105F/89M  Frailty definition: Fried phenotype with modifications: unintentional weight loss within previous six months recorded within medical records, an exhaustion question from the Patient Health Questionnaire-9 and the Short Form-12 physical score for low activity.  Baseline frailty: non-frail 75, pre-frail 87, frail 32 | 6, 12 months 1. Primary outcome: hospitalisations and emergency department visits****  2. Transition to a worse frailty state (Fried criteria) | Slightly higher transitions from pre-frail to non-frail in usual care compared to telemonitoring (12/38 (32%) vs 9/35 (26%)) and from pre-frail to frail (6/38 (16%) vs 3/35 (9%)) between baseline and 6 months. Similar numbers remained pre-frail (20/38 (53%) vs 21/35 (60%)). 2 telemonitoring and 9 usual care deaths. |

*Further data supplied from the authors.

**n=32 analysed in original paper from (period 1 exercise and period 2 exercise groups combined vs 1^st^ period control). N=16 results obtained from authors for meta-analysis.

*** as reported in this paper, protocol states this was a secondary outcome

****reported in separate paper, without frailty stratification

Abbreviations: ADL= activities of daily living; ANOVA = analysis of variance; CI = confidence interval; dL = decilitre; F=female; FSQ = functional status questionnaire; kg = kilograms; M=male; m = metre; m/s = metres per second; mPPT: modified physical performance test; SPPB = Short Physical Performance Battery; VO_2peak_ = peak oxygen consumption; yrs=years.
