# Supplementary material for: Health promotion interventions for community-dwelling older people with mild or pre-frailty: a systematic review and meta-analysis
Source: BMC Geriatr. 2017 Jul 20;17:157. doi: 10.1186/s12877-017-0547-8 (PMC5520298; doi:10.1186/s12877-017-0547-8)
Supplement: Supplementary file 2 — Table of ongoing studies identified in this review. (DOCX 19 kb) [file 12877_2017_547_MOESM2_ESM.docx]

**Supplementary file 2: Ongoing studies**

**Studies identified from searches 6.6.16 of Clinicaltrials.gov search, UK Clinical Trials Gateway and HTA database plus database searches.**

| **Pre-frail populations** | | | | | |
| --- | --- | --- | --- | --- | --- |
| **Title** | **Lead author** | **Status** | **Pre-frailty criteria** | **Intervention** | **Identifier or location** |
| Clinical and Economic Assessment of a Pre-frail Screening Program | M Serra-Prat  Spain | Completed, published 7.1.17 | Pre-frailty using Fried criteria | Nutritional assessment and physical activity programme vs usual care | Serra-Prat et al. *Age and Ageing.* 2017. 0:1-7.^44^ |
| Training and de-training effects: One year follow-up of a 3-month resistance exercise program in the pre-frail elderly | P Lin | Completed, published as conference abstract | Pre-frailty (criteria not reported) | Muscle resistance training vs control (not specified) | Lin et al. *Physiotherapy.* 2015. 101: eS882. |
| The Effect of SOD Enzyme on Frailty and HRQOL Among Indonesian Pre-frail Elderly: A Double Blind Randomized Controlled Trial | S Setiati  Indonesia | Recruiting | Pre-frailty on FI-40 item questionnaire | Superoxide Dismutase enzyme and Gliadin supplement vs placebo | NCT02753582  Clinicaltrials.gov |
| Resistance Training to Optimize Health in Pre-frail Older Adults | A Tang  Canada | Recruiting | Pre-frailty (criteria unclear) | Higher intensity resistance training vs lower intensity resistance training | NCT02593084  Clinicaltrials.gov |
| Home-based health promotion for vulnerable older people | K Walters  UK | Completed | “Mild frailty” on Clinical Frailty Scale | Home-based multidimensional health promotion and behaviour change intervention vs treatment as usual | ISRCTN11986672  UK Clinical Trials Gateway |

| **Pre-frail and frail populations*** | | | | | |
| --- | --- | --- | --- | --- | --- |
| **Title** | **Authors** | **Status** | **Pre-frailty criteria** | **Intervention** | **Identifier or location** |
| Immune Benefits of WGP in Elderly | Nestle  Germany | Completed | Frail or pre-frail (Fried criteria) | Food fibres dietary supplement plus influenza vaccine vs placebo (maltodextrin) plus influenza vaccine | NCT02262091  Clinicaltrials.gov |
| Effectiveness of a Program Using Video Games Associated With Conventional Physiotherapy in Physical Functioning in Frail Elderly Compared to Conventional Physiotherapy | M Perracini  Brazil | Recruiting | Frail or pre-frail (Fried criteria) | Exergames and conventional physiotherapy vs conventional physiotherapy | NCT02333214  Clinicaltrials.gov |
| Effects of Community Health Programs by Nurses for Older Adults | L Huang  Taiwan | Completed | Fried frailty criteria: Frail or pre-frail | Comprehensive community nursing care, including physical activity training, community resources referrals, health education and health promotion vs usual care | NCT01972958  Clinicaltrials.gov |

*insufficient information to determine whether pre-frail results would be reported separately

**Updated search 10.1.17 of Clinicaltrials.gov, UK Clinical Trials Gateway and HTA database**

| **Pre-frail and frail populations*** | | | | | |
| --- | --- | --- | --- | --- | --- |
| **Title** | **Authors** | **Status** | **Pre-frailty criteria** | **Intervention** | **Identifier or location** |
| Exercise Intervention to Reverse Frailty (ERF) | G Jones  Canada | Recruiting | Women with gait speed 1-1.5m/s and pre-frail according to the CHS and "Vulnerable" and/or "Mildly Frail" according to the CFS | Exercise intervention (resistance, aerobic, flexibility and balance) vs usual activity | NCT02952443  Clinicaltrials.gov |
| Home-Based Technologies Coupled to Teleassistance Service in the Elderly (DOMOLIM) | T Dantoine  France | Recruiting | Frail or pre-frail (Fried criteria) | Home automation pack with teleassistance vs teleassistance only | NCT01697553  Clinicaltrials.gov |
| Implementing Resistance Exercise to Reduce Frailty for Older Adult Medicaid Waiver Recipients | M Danilovich  USA | Not yet open for recruitment | Frail or pre-frail (SHARE-FI) | Resistance exercise vs usual care | NCT02942992  Clinicaltrials.gov |
| A person-centred approach to health promotion for persons 70+ who have migrated to Sweden: promoting aging migrants’ capabilities | S Gustafsson  Sweden | Completed | Eight physical frailty indicators assessed as outcomes in community-dwelling migrants aged 70+ born in Finland or the Balkan Peninsula not dependent on informal or formal help in daily activities. | Senior meetings (multidimensional, delivered by nurses, physiotherapists, occupational therapists and social workers) followed by a home visit vs usual care | NCT01841853  Clinicaltrials.gov |

*insufficient information to determine whether pre-frail results would be reported separately
